# Postmortem Interval Leads to Loss of Disease-Specific Signatures in Brain Tissue

**DOI:** 10.1523/ENEURO.0505-24.2025

**Published:** 2025-03-07

**Authors:** Kimberly C. Olney, Katelin A. Gibson, Mika P. Cadiz, Negin Rahimzadeh, Vivek Swarup, John D. Fryer

**Affiliations:** ^1^Department of Neuroscience, Mayo Clinic, Scottsdale, Arizona 85259; ^2^Translational Genomics Research Institute (TGen), Phoenix, Arizona 85004; ^3^Department of Neurobiology and Behavior, University of California, Irvine, California 92697; ^4^Neuroscience Graduate Program, Mayo Clinic Graduate School of Biomedical Sciences, Scottsdale, Arizona 85259

**Keywords:** gene expression, postmortem interval, single nucleus

## Abstract

Human brain banks are essential for studying a wide variety of neurological and neurodegenerative diseases, yet the variability in postmortem interval (PMI)—the time from death to tissue preservation—poses significant challenges due to rapid cellular decomposition, protein alterations, and RNA degradation. Furthermore, the postmortem transcriptomic alterations occurring within distinct cell types are poorly understood. In this study, we analyzed the effect of a 3 h postmortem interval on single-nucleus RNA signatures in the brains of wild-type (WT) and PS19 mice, a common model of tauopathy. We observed that basic quality control metrics (such as the number of genes and reads per cell), total nuclei counts, and RNA integrity number (RIN^e^) remained consistent across all samples, regardless of PMI or genotype. However, a 3 h PMI diminished the number of genes differentially expressed between PS19 and WT mice, suggesting an impact of delayed processing on the detection of disease-specific transcriptomic signatures. When directly comparing 3 h PMI versus freshly harvested 0 h mouse brains, we identified genes upregulated in neurons and interneurons involved in DNA repair, immune response, and stress pathways. Furthermore, genes that were altered in non-neuronal cell types at 3 versus 0 h PMI were associated with cell–cell adhesion processes. These findings highlight the effects of PMI on single-nucleus transcriptional changes that may dampen the true changes in cellular states in banked brain tissues.

## Significance Statement

This study investigates how postmortem interval (PMI)—the time between death and tissue preservation—affects gene expression in brain cell types using single-nucleus RNA sequencing. By comparing brain samples collected immediately and 3 h postmortem in mice, we found that PMI can obscure disease-related gene expression changes, especially in neurons. These findings underscore the importance of accounting for PMI in studies of neurodegenerative disease using human brain banks.

## Introduction

Studies of neurological conditions benefit immensely from using “banked” or frozen human brain samples to assess molecular changes driven by disease, as mouse models often fail to capture the full spectrum of disease phenotypes. However, postmortem interval (PMI), the time between death and brain preservation or tissue processing, varies widely within and across brain banks and may confound the analysis of human samples. Research on the impact of PMI on gene expression has shown varying effects depending on tissue type and preservation method (Zhu et al., 2017; Scott et al., 2020). In human postmortem tissue, PMI altered the transcriptome in the central nervous system (CNS) and blood, dysregulating pathways involved in immune response and proteolysis, and cell cycle, respectively (Zhu et al., 2017). Other studies have suggested that PMI minimally affects gene expression, with factors such as brain pH, sex, and age at death being more influential (Preece and Cairns, 2003), and that RNA integrity is generally maintained across different brain regions despite long PMIs (Ervin et al., 2007). PMI effects may also be cell type specific; a bulk transcriptomic analysis of human neocortex tissue surgically resected from epilepsy patients revealed a rapid decline in neuronal and activity-dependent gene expression in tissue processed 24 h after resection compared with freshly resected tissue (Dachet et al., 2021). In contrast, glia-specific gene expression increased (Dachet et al., 2021). These studies collectively suggest that while PMI can influence gene expression, the extent and nature of this impact are variable and dependent on specific tissue.

We extended this work by providing an in-depth, single-nucleus resolution characterization of PMI-induced transcriptional changes in the brains of 37-week-old (∼9.25-month-old) wild-type (WT) and tau P301S (PS19) mice, a model of tauopathy commonly used in Alzheimer's disease studies (Yoshiyama et al., 2007; Iba et al., 2013). Tauopathies are neurodegenerative disorders characterized by abnormal tau protein aggregations that impair normal neuronal function. The median lifespan of these mouse models is 27–29 months; thus, a 37-week-old mouse corresponds roughly to a 35-year-old human, representing mature adulthood. Using mouse models, we aimed to identify postmortem changes that are not confounded by age, diet, or genetic background. Our study design was explicitly structured to mirror conditions as closely as possible to the human postmortem interval (PMI). When individuals agree to donate their brain to a brain bank, their brain often experiences a delay in retrieval due to procedures and transport, including going to a morgue before an autopsy team can remove their brain. The brain cools down naturally within the intact skull; thus, in this study, 3 h PMI brains remained intact in the skull until processing. This delay can impact tissue quality and composition in ways that are difficult to replicate by removing the brain and holding it outside the skull.

## Materials and Methods

### Wild-type and tau mouse brains

**Figure 1.**
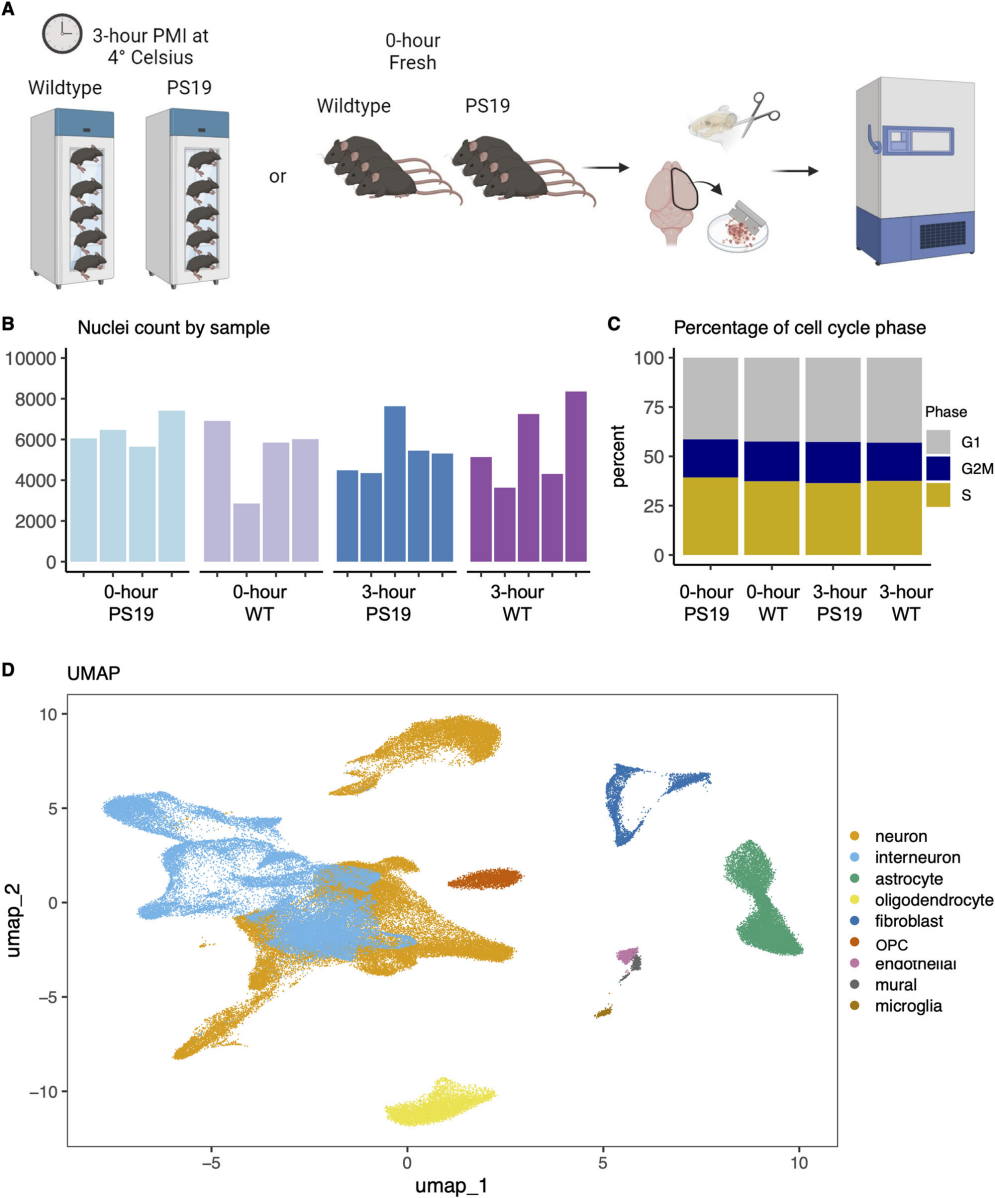
Single-nucleus quality metrics do not differ between fresh 0 and 3 h postmortem interval (PMI) mice. ***A***, Schematic representation of the experimental setup. Single-nucleus RNA libraries were generated from the brains of 37-week-old C57BL/6J wild-type (*N* = 9; WT) and tau P301S (*N* = 9; PS19) mice. Samples were collected immediately post-killing (*N* = 8; 0 h PMI) or 3 h postmortem (*N* = 10; 3 h PMI). ***B***, Bar graph showing the total nuclei count per group, categorized by PMI and genotype. ***C***, The percentage graph illustrating the distribution of nuclei across different cell cycle phases (G1, S, G2/M) is similar across all groups. ***D***, UMAP visualizing the clustering of annotated cell types. Sample information and RNA integrity number (RIN^e^) in Extended Data Table 1-1. Verification of the expression of human *MAPT* in PS19 mice and the genetic sex of the mice were investigated, as shown in Extended Data Figure 1-1. Group and individual quality control metrics are shown in Extended Data Figure 1-2. Extended Data Figure 1-3 further shows cell type abundance among samples. Extended Data Figure 1-4 displays the expression of cell type markers within each identified cell type. Extended Data Figure 1-5 displays the principal component analysis (PCA) of the aggregated expression values at the sample and cell type level, which shows a clear delineation of the major cell types.

We used *N* = 9 wild-type (WT) C57BL/6J and *N* = 9 tau P301S (PS19) mice (Yoshiyama et al., 2007; Iba et al., 2013) at 37 weeks of age in this study. PS19 mice were bred with pure C57Bl/6J background after >10 generations to ensure that the PS19 genetic background is nearly identical to the C57Bl/6J strain, apart from the transgene (P301S tau). All studies were conducted in accordance with the National Institutes of Health Guide for the Care and Use of Laboratory Animals under an approved protocol from the Mayo Clinic Institutional Animal Care and Use Committee. Mice were killed by carbon dioxide overdose followed by cervical dislocation either 3 h postmortem or immediately (fresh “0 h”) before processing (Fig. 1*A*). For the 0 h PMI group, *N* = 4 WT (2 males and 2 females) and *N* = 4 PS19 (3 males and 1 female) mice were used. In the 3 h PMI group, *N* = 5 WT mice (2 males and 3 females) and *N* = 5 PS19 (3 males and 2 females) mice were utilized (Extended Data Table 1-1). For the 3 h PMI group, the whole corpses of the killed mice were placed intact at 4°C for 3 h before subsequently removing the brain for further processing (Fig. 1*A*). The brain cools down naturally within the intact skull. This delay can impact tissue quality and composition in ways that are difficult to replicate by removing the brain and holding it outside the skull. The study includes balanced groups across genotypes (WT and PS19) and postmortem intervals (0 and 3 h), ensuring sufficient representation for each condition and an adequate sample size to detect biological differences while minimizing unnecessary animal use. Mice were randomly selected to be either in the 0 or 3 h PMI group while maintaining roughly equal representation of both sexes and genotypes.

10.1523/ENEURO.0505-24.2025.f1-1 Figure 1-1 **Sample genotype and sex verification.** Log normalized gene expression of **A)** mouse *Mapt*, **B)** human *MAPT*, mouse **C)** *Xist*, and **D)** *Uty*. Download Figure 1-1 TIF file.

**Figure 2.**
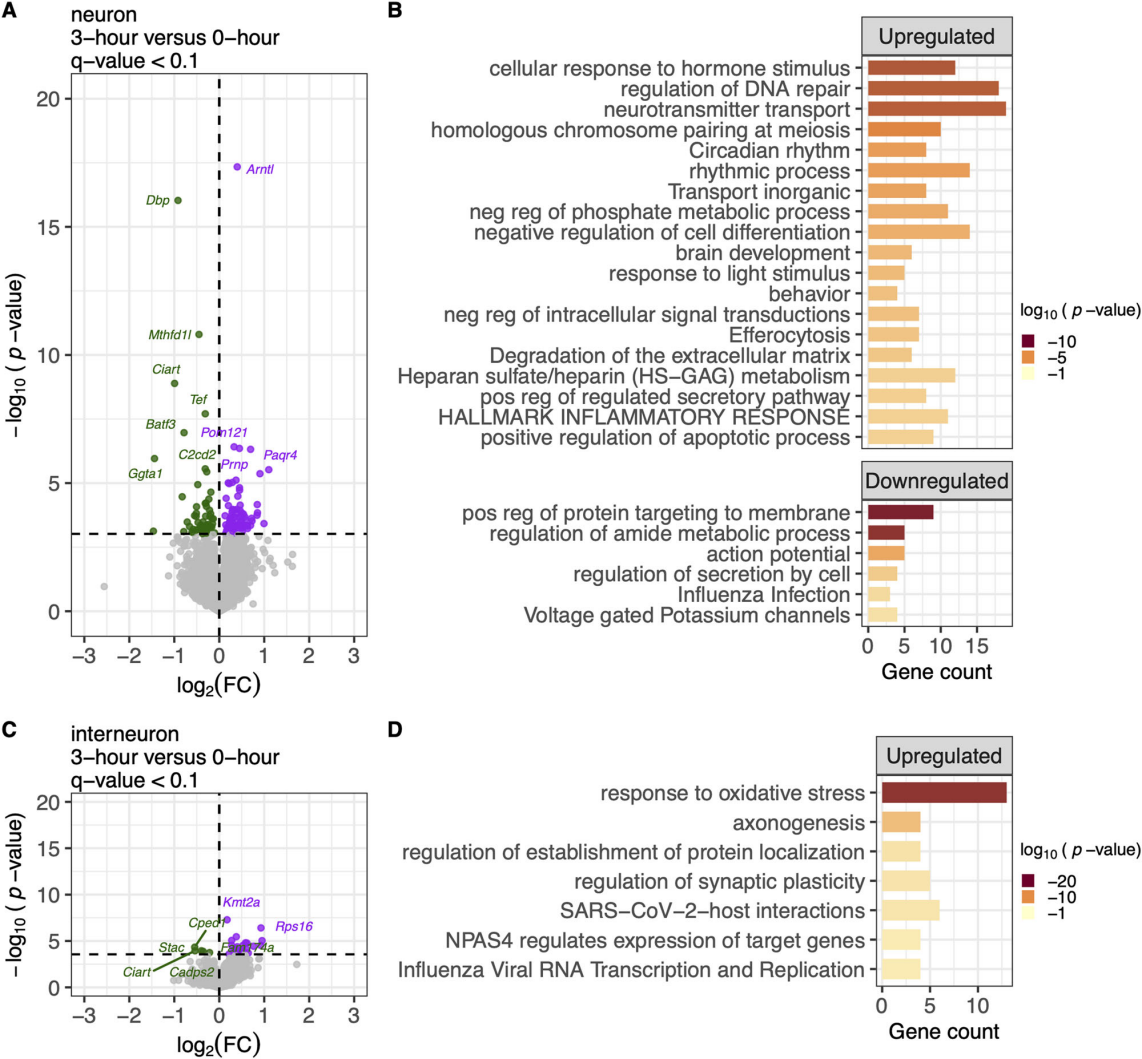
Genes differentially expressed between 3 h PMI and 0 h fresh samples within the neuron cell types are involved in immune response and stress pathways. ***A***, Volcano plot of differentially expressed genes (DEGs) in neuron cell type, comparing 3 h PMI, *N* = 10 versus fresh 0 h, *N* = 8 samples (total *N* = 18). Downregulated genes (log2 fold change < 0, *q* < 0.1) are shown in green, upregulated genes (log2 fold change > 0, *q* < 0.1) in purple, and nonsignificant genes (*q* ≥ 0.1) in gray. ***B***, Gene ontology (GO) analysis showed an upregulation of cellular response, circadian rhythm, inflammatory response, and positive regulation of the apoptotic process. Downregulated genes are enriched in regulating metabolic processes, cell secretion, and influenza infection. The *x*-axis is the GO term, and the *y*-axis is the −log10 *p* value. ***C***, ***D***, Similarly, genes upregulated within the interneuron cell type between 3 h PMI and fresh 0 h are enriched in response to oxidative stress and regulation of synaptic plasticity.

10.1523/ENEURO.0505-24.2025.f1-2 Figure 1-2 **Single nucleus quality metrics do not differ among mice. A)** Violin plots illustrating primary quality control (QC) metrics, including the number of counts per nucleus (nCount), detected features per nucleus (nFeature), cell complexity (the log_10_ ratio of nFeature to nCount), and the percentage of mitochondrial gene expression (percent mt) for PS19 0-hour (N = 4), WT 0-hour (N = 4), PS19 3-hour (N = 5), and WT 3-hour (N = 5) mice. Quality control metrics for each mouse for **B)** nCount, **C)** nFeature, **D)** cell complexity, and **E)** percent mt. Download Figure 1-2 TIF file.

**Figure 3.**
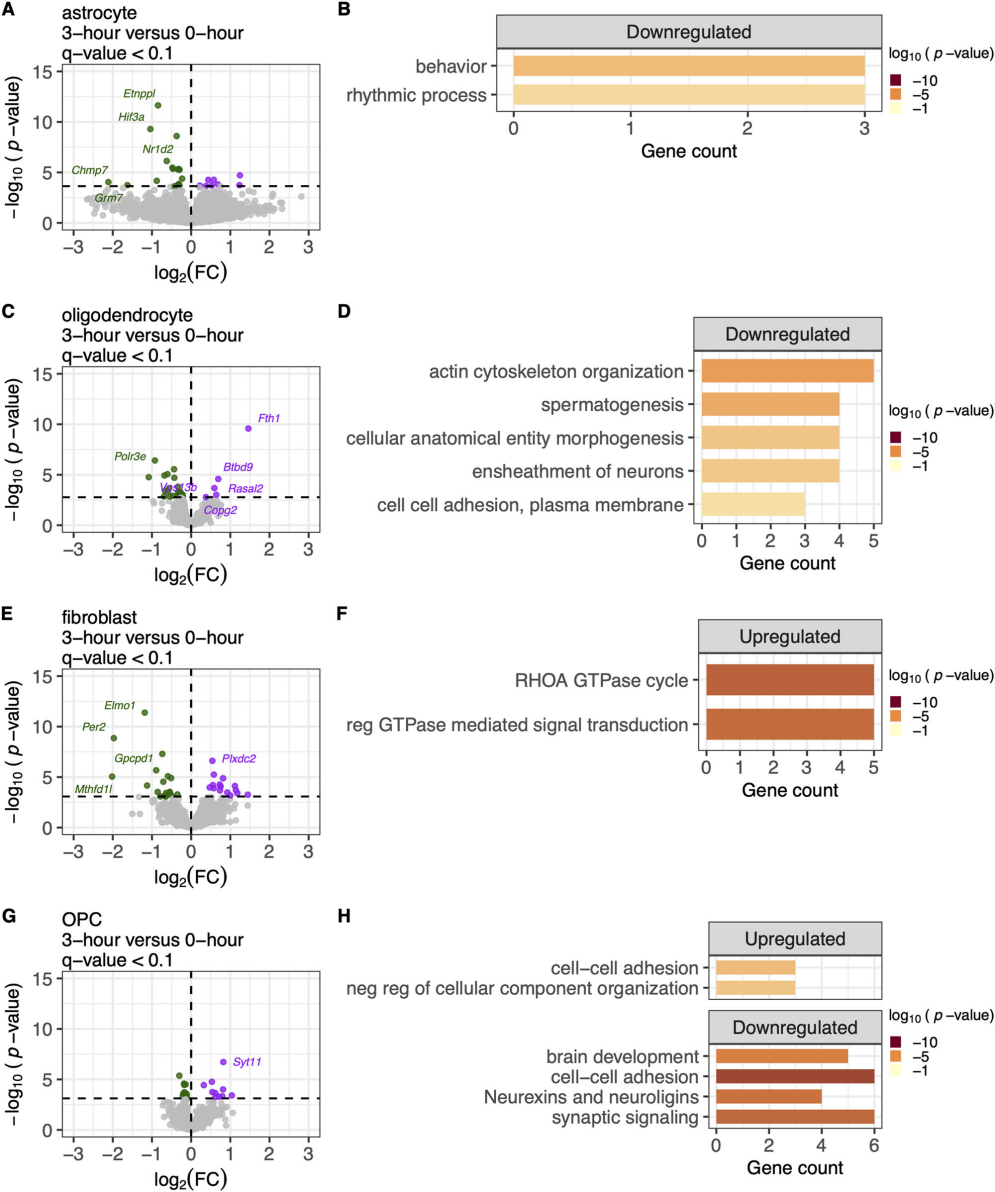
Genes differentially expressed between 3 h PMI and 0 h fresh samples within the glial cell types are involved in rhythmic processes and cell–cell adhesion pathways. ***A***, Volcano plot of differentially expressed genes (DEGs) in astrocyte cell type, comparing 3 h PMI, *N* = 10 versus fresh 0 h, *N* = 8 samples (total *N* = 18). Downregulated genes (log2 fold change < 0, *q* < 0.1) are shown in green, upregulated genes (log2 fold change > 0, *q* < 0.1) in purple, and nonsignificant genes (*q* ≥ 0.1) in gray. ***B***, Gene ontology (GO) analysis shows the downregulation of behavior and rhythmic processes. The *x*-axis is the gene count contributing to the enrichment pathways listed on the *y*-axis. The color of the bar indicates the −log10 *p* value. ***C–F***, Similarly, genes downregulated or upregulated within the other glial cell types, including oligodendrocytes, fibroblast, and OPC are enriched in cell–cell adhesions, GTPase cycle, brain development, and synaptic signaling. Extended Data Figure 3-1 displays the transcriptomic effect of PMI 3 versus 0 h PMI for endothelial, mural, and microglia cell types.

10.1523/ENEURO.0505-24.2025.f1-3 Figure 1-3 **Relative abundance of cell types among samples. A)** the total nuclei count for each cell type is shown for both the 0-hour and 3-hour post-mortem interval (PMI) sample groups. **B)** the percentage of each cell type is presented across different genotypes (PS19 or WT) and PMI durations (0-hour or 3-hour). **C)** The proportion of each cell type within individual samples is illustrated, providing a detailed view of the distribution across all samples. Download Figure 1-3 TIF file.

**Figure 4.**
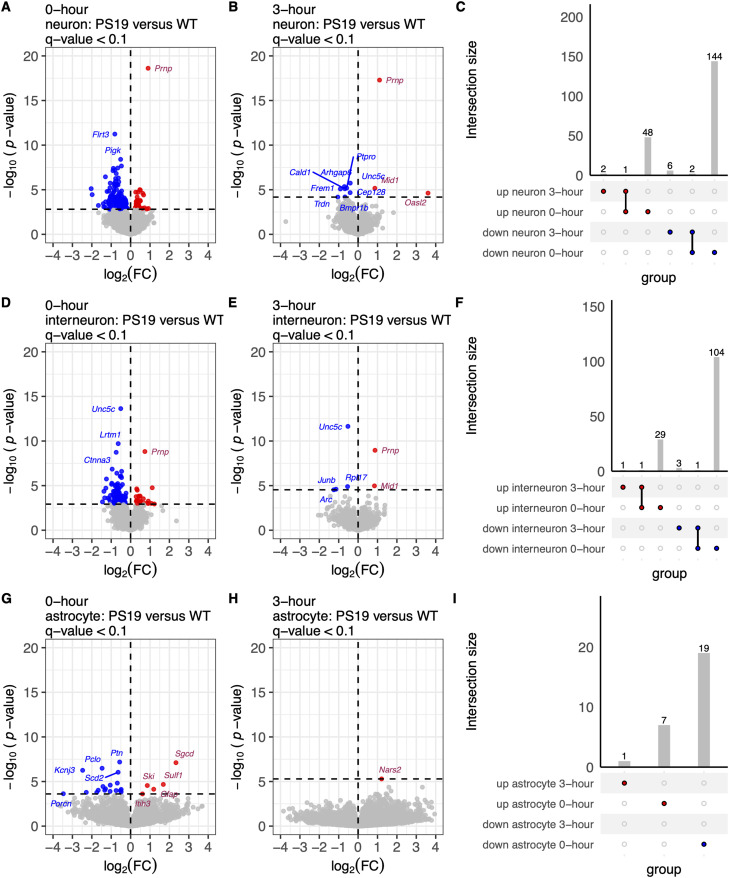
Reduced PS19 versus WT differential expression signal at the 3 h PMI compared with 0 h fresh samples. ***A***, Volcano plot of differentially expressed genes (DEGs) in neuron cell type, comparing PS19 (*N* = 4) versus WT (*N* = 4) in fresh 0 h samples (total *N* = 8). Downregulated genes (log2 fold change < 0, *q* < 0.1) are shown in blue, upregulated genes (log2 fold change > 0, *q* < 0.1) in red, and nonsignificant genes (*q* ≥ 0.1) in gray. ***B***, Volcano plot of DEGs in neuron cell types, comparing PS19 (*N* = 5) versus WT (*N* = 5) in 3 h PMI samples (total *N* = 10). ***C***, UpSet plot depicting shared and unique DEGs between 0 and 3 h PMI analyses. Panels ***D–F*** present the corresponding analyses for interneurons and panels ***G–I*** for astrocytes. Extended Data Figure 4-1 displays the gene ontology (GO) enrichment analysis of upregulated and downregulated genes in PS19 versus WT at 0 and 3 h postmortem intervals (PMI).

10.1523/ENEURO.0505-24.2025.f1-4 Figure 1-4 **Bubble plot showing brain cell type markers.** The Y-axis represents different cell types, while the X-axis displays the corresponding biomarker genes. The size of each bubble indicates the percentage of nuclei within a given cell type that expresses that gene and the color represents the average log_2_ expression level. Download Figure 1-4 TIF file.

10.1523/ENEURO.0505-24.2025.f1-5 Figure 1-5 **Principal component analysis (PCA) of aggregated expression values at the sample and cell type level.** Clear delineation of the major cell types. Download Figure 1-5 TIF file.

10.1523/ENEURO.0505-24.2025.t1-1 Table 1-1 **Metadata.** Sample ID, genotype PS19 tau or wildtype (WT), sex, post-mortem interval (PMI) in hours, and RNA integrity number (RIN^e^). Download Table 1-1. Metadata., DOCX file.

### Tissue dissociation and preparation

Brains were extracted from mice, bilaterally dissected, and an entire hemiforebrain was finely chopped, aliquoted into three tubes, and snap-frozen using dry ice for single-nucleus RNAseq (snRNAseq). Total RNA was extracted from frozen mouse brain tissue using the Qiagen RNeasy Plus Mini Kit, following the manufacturer's protocol. RNA integrity was assessed on an Agilent TapeStation using the RNA ScreenTape kit to obtain RIN^e^ values (Extended Data Table 1-1). Nuclei were isolated using Miltenyi Nuclei Extraction Buffer (Miltenyi Biotec, #130-128-024), followed by Miltenyi debris removal. Promega RNase Inhibitor (Promega, #PR-N2115) was included in all buffers and wash steps at a final concentration of 0.2 U/µl to prevent RNA degradation. Tissue samples were placed in a gentleMACS C Tube (Miltenyi Biotec, #130-093-237) containing 2 ml of precooled Nuclei Extraction Buffer supplemented with RNase Inhibitor (0.2 U/µl). The samples were dissociated using the gentleMACS Octo Dissociator with Heaters (Miltenyi Biotec, #130-096-427), running the 4C_nuclei_1 protocol. The resulting suspension was passed through a precooled 100 μm strainer to remove debris and large particles and washed with 2 ml of precooled Nuclei Extraction Buffer supplemented with RNase Inhibitor. The nuclei suspension was centrifuged at 300 × *g* for 10 min at 4°C. After centrifugation, the supernatant was aspirated, and the pellet was resuspended in 3,100 μl of DPBS and 900 μl of debris removal solution (Miltenyi Biotec, #130-109-398). The suspension was transferred to a 15 ml tube and gently overlaid with 4 ml of cold DPBS. The nuclei were centrifuged at 3,000 × *g* for 10 min at 4°C with full acceleration and 50% brake. This produced three dist115 inct phases, of which the top two were carefully aspirated and discarded. The remaining solution was filled to a final volume of 15 ml with cold DPBS and gently inverted three times. A second centrifugation was performed at 1,000 × *g* for 10 min at 4°C with full acceleration and brake. The supernatant was then aspirated completely, and the nuclei were resuspended in 0.04% BSA in PBS for counting and further processing of single-nucleus GEM generation using the 10x Genomics Chromium Controller.

### Library preparation and sequencing

Following nuclei isolation and counting, ∼6,000 nuclei per sample were processed using the 10X Genomics 3′ Single Cell Gene Expression Kit v3.1 (10X Genomics, #1000121), following the manufacturer's protocol. Library preparation was performed over 2 consecutive days to reduce batch effects. Nuclei were diluted to the optimal concentration and loaded onto a 10x Genomics Chromium Single Cell G Chip for Gel Bead-In Emulsion (GEM) generation. After GEM generation, reverse transcription (GEM-RT) was performed, and samples were held at 4°C until all were ready for further processing. Since only eight samples could be run per chip, GEM-RT was synchronized across batches. Upon completion, GEMs were broken to release barcoded cDNA. Post-GEM cleanup utilized Dynabeads MyOne Silane to separate cDNA from oil and reagents. The cDNA was amplified via PCR, purified with SPRIselect beads, and quality controlled using the Agilent TapeStation. Following cDNA fragmentation, end repair, and A-tailing, a second SPRIselect-based size selection was performed. Adapters were ligated, and indexing was completed by PCR, followed by double-size selection. Final libraries were quantified and quality checked with the Agilent TapeStation to ensure libraries met the quality control standards for sequencing as suggested by 10x Genomics. All libraries passed quality control. Libraries were then sequenced on an Illumina PE150 flow cell for downstream analysis.

### Single-nucleus RNAseq data processing

Samples were aligned to the mouse GRCm39 reference genome via Cell Ranger. The mouse GRCm39 reference genome was obtained from the 10x Genomics downloads page. The human *MAPT* gene was integrated into the mouse reference genome to verify the expression of human *MAPT* in PS19 mice (Extended Data Fig. 1-1). Sex verification of samples was conducted by examining the expression levels of *Uty* and *Xist* (Extended Data Fig. 1-1; Olney et al., 2020). Ambient RNA was identified and removed using CellBender (Fleming et al., 2023). Post-CellBender quality control checks and filtering were performed with the following criteria: nuclei with counts per nucleus (nCount) between 500 and 35,000, detected genes per nucleus (nFeature) between 300 and 8,000, and complexity, defined as log10(nFeature/nCount), above 0.80 were retained. Additionally, cutoffs were applied for the percent of reads aligning to mitochondrial (1%), hemoglobin (0%), ribosomal (1%), and choroid plexus genes, *Ttr*, *Folr1*, and *Prnpl*, (0%) to avoid unintentional biases (Olney et al., 2022). After initial quality control processing, genes with low expression were excluded, retaining only those with at least one count in 10 nuclei, and any residual mitochondrial encoded genes were removed. Data normalization and scaling were performed using the SCTransform function in Seurat V5 (Hafemeister and Satija, 2019). Cell type annotation was conducted using established brain cell type markers (McKenzie et al., 2018) and the top genes from Seurat's FindMarkers function. Doublets were identified and removed using the DoubletFinder package (McGinnis et al., 2019). After doublet removal, nuclei were reclustered and reannotated to ensure accurate identification of cell types (Extended Data Fig. 1-2).

### Differential expression

To mitigate false positives and better account for within-sample correlations, we performed differential expression analysis with pseudobulking. This approach aggregates expression values at the sample and cell type level. Pairwise differential expression analysis was then performed using the DESeq2 package (Love et al., 2014). Prior studies have shown varied performance across differential expression tools, highlighting a trade-off between detecting true positives and maintaining precision. DESeq2 pseudobulk was repeatedly reported as being well balanced and robust for small sample size (Jaakkola et al., 2017; Wang et al., 2019; Squair et al., 2021; Zimmerman et al., 2021; Murphy and Skene, 2022; Li et al., 2023). This analysis focused on protein-coding genes, excluding those with mean expression levels below the first quartile for each cell type. Depending on the specific comparison—whether between genotypes or PMIs—covariates genotype, sex, and PMI were included in the model. When comparing PS19 versus WT within a PMI (fresh 0 or 3 h), only sex was included as a covariate in the model. Genes were considered differentially expressed using a Benjamini–Hochberg false discovery rate (FDR) of *q* value of <0.1.

### Statistics

All statistical analyses were performed using R (version 4.3.2). To assess normality, the Shapiro–Wilk test was used for each group. For comparisons between groups with normally distributed data, one-way analysis of variance (ANOVA) tests was conducted (Table 1). A comparison of RIN^e^ values between fresh 0 and 3 h PMI was conducted using a two-sample *t* test (Table 1). Differences in cell type proportions across groups were tested using chi-square tests. Differential gene expression analysis was performed with DESeq2 (version 1.42.1), which estimates expression changes across conditions while accounting for biological variance. DESeq2 was used to identify genes with significant changes in expression between postmortem intervals (PMI) and genotypes (PS19 vs WT); significance thresholds were set at *α* = 0.1.

**Table 1.** Statistical summary

	Data structure	Type of test	Test statistic	*p* value	*N* (mice)
Figure 1*B*	Normal distribution	One-way ANOVA	*F*_(3,14)_ = 0.36	*p* = 0.786	18
Figure 1*C*	Contingency table	Chi-square test of independence	*X*^2^ = 0.06, df = 6	*p* = 1	18
Figure 1-4	Contingency table	Chi-square test of independence	*X*^2^ = 4.26, df = 24	*p* = 1	18
Table 1	Normal distribution	Two-sample *t* test	*t* = 0.893, df = 16	*p* = 0.385	18

The Shapiro–Wilk test was applied to each group to assess normality. A one-way analysis of variance (ANOVA) was conducted to compare groups with normally distributed data. A comparison of RIN^e^ values between fresh 0 and 3 h postmortem intervals (PMI) was performed using a two-sample *t* test. Differences in cell type proportions across groups were analyzed using chi-square tests.

### Data and code availability

Raw data are deposited in the Sequence Read Archive (SRA), accessible via PRJNA1171828. The code used for analysis is available at https://github.com/olneykimberly/PMI/. Interactive Shiny app to explore genes of interest: https://fryerlab.shinyapps.io/single_nucleus_PMI/.

## Results

### Quality control metrics and cell cycle phase do not significantly vary between genotypes or PMI

We first investigated RIN^e^ values to determine if a delayed PMI resulted in lower RIN values. There was no difference in mean RIN^e^ values between fresh 0 and 3 h PMI (*t*_(16)_ = 0.893, *p* = 0.385). Key quality control metrics, including the number of counts per nucleus (nCount), detected genes per nucleus (nFeature), cell complexity log10(nFeature/nCount), and the percentage of reads aligned to mitochondrial genes, were also evaluated. These metrics were consistent across all groups, regardless of postmortem interval (0 or 3 h) or genotype (PS19 or WT; Extended Data Fig. 1-2). The total nuclei count per sample after quality filtering ranged from 3,353 to 9,905 nuclei (Fig. 1*B*). The mean nuclei count per group categorized by PMI and genotype (PS19 or WT) showed no significant differences between the experimental conditions (ANOVA *F*_(3,14)_ = 0.36, *p* = 0.786; Fig. 1*B*). The similarity in QC metrics between fresh 0 and 3 h PMI groups suggests that the overall data quality is maintained despite the postmortem delay, supporting the reliability of the sequencing results under these conditions. Genes associated with the cell cycle phase were previously reported to be downregulated in relation to postmortem interval in whole blood but not in brain tissue (Zhu et al., 2017). Our data showed no significant differences in cell cycle phase distributions among the groups categorized by PMI and genotype (chi-square, *X*^2^ = 0.06, df = 6, *p* = 1; Fig. 1*C*). Nuclei were distributed similarly across the G1, S, and G2/M phases in all conditions (Fig. 1*C*).

### Cell type proportions are consistent across genotypes and PMI

After data cleaning, clustering, and cell type annotation with Seurat, we found that neurons and interneurons were the most prevalent cell types in our data (Fig. 1*D*, Extended Data Fig. 1-3), consistent with other single-nucleus RNAseq data where neurons typically outnumber other cell types (Saunders et al., 2018). Neurons and interneurons were the predominant cell types across all samples (Extended Data Fig. 1-3). Interneurons showed expression of *Gad1* and *Gad2*, which was not observed within the neuron cluster (Extended Data Fig. 1-4). Furthermore, when aggregate expression values at the sample and cell type level were followed by principal component analysis (PCA), we observed a clear separation between the neurons and interneurons (Extended Data Fig. 1-5). While the proportions of cell types showed some variation among genotypes and PMI, there were no significant differences between groups (chi-square, *X*^2^ = 4.26, df = 24, *p* value = 1). This consistency in cell type proportions across different conditions suggests that neither genotype nor a 3 h PMI significantly alters nuclei capture with this method of single-nucleus RNAseq preparation.

### A 3 h PMI induces robust cell type-specific transcriptional alterations

Using pseudobulk DESeq2 differential expression analysis, we identified numerous cell type-specific transcriptional alterations at 3 versus 0 h postmortem. These changes were most pronounced in neurons (Fig. 2*A*) and interneurons (Fig. 2*C*) but were also present in astrocytes (Fig. 3*A*), oligodendrocytes (Fig. 3*C*), fibroblasts (Fig. 3*E*), and oligodendrocyte progenitor cells (OPC; Fig. 3*G*). In neurons, we identified 79 upregulated and 53 downregulated differentially expressed genes (DEGs; *q* value <0.1) at the 3 versus 0 h PMI (Fig. 2*A*). The 79 upregulated genes were associated with pathways related to regulating DNA repair, hallmark inflammatory response, and action potential (Fig. 2*B*). The 53 downregulated genes were enriched in pathways associated with regulating amine metabolic process, action potential, and regulation of secretion by cell (Fig. 2*B*). Similar patterns were observed in interneurons, with 26 upregulated and 5 downregulated genes (*q* value <0.1; Fig. 2C) involved in oxidative stress response and synaptic plasticity regulation (Fig. 2*D*). These findings underscore the sensitivity of neuronal gene expression to postmortem delay and emphasize the importance of accounting for PMI, as it can influence the expression of genes involved in pathways often associated with disease state.

10.1523/ENEURO.0505-24.2025.f3-1 Figure 3-1 **The transcriptomic effect of PMI 3-hour versus 0-hour PMI for endothelial, mural, and microglia cell types. A)** Volcano plot of differentially expressed genes (DEGs) in endothelial cell type, comparing 3-hour PMI (N = 10) versus fresh 0-hour (N = 8) samples (total N = 18). Downregulated genes (log2 fold change < 0, q < 0.1) are shown in green, upregulated genes (log_2_ fold change > 0, q < 0.1) in purple, and non-significant genes (q ≥ 0.1) in gray. Repeated for **B)** mural and **C)** microglia. Download Figure 3-1 TIF file.

Despite the lower number of nuclei in glial cell types, there were still apparent differences in gene expression between 3 and 0 h PMI samples (Fig. 3, Extended Data Fig. 3-1). Astrocytes, for instance, had significant changes, with 8 upregulated and 18 downregulated DEGs (Fig. 3*A*). The downregulated genes were enriched in pathways related to behavior and rhythmic processes (Fig. 3*B*). Oligodendrocytes show a similar pattern, with 5 upregulated and 24 downregulated DEGs (Fig. 3*C*). The downregulated oligodendrocyte genes were enriched in cell–cell adhesion pathways (Fig. 3*D*). Fibroblasts also displayed significant gene expression changes, with 8 upregulated and 18 downregulated DEGs (Fig. 3*E*). Upregulated genes in fibroblasts were enriched in pathways related to RHOA GTPase cycle and the regulation of GTPase-mediated signal transduction (Fig. 3*F*). In OPCs, 10 upregulated and 12 downregulated DEGs were observed (Fig. 3*G*). Upregulated genes were enriched in pathways involving cell–cell adhesion and the negative regulation of cellular component organization (Fig. 3*H*). In contrast, downregulated genes were associated with brain development, cell–cell adhesion, neurexins/neuroligins, and synaptic signaling (Fig. 3*H*). The cell–cell adhesion pathway is notably enriched across multiple cell types, which may indicate altered intercellular interactions due to postmortem delay.

As anticipated, fewer DEGs were detected in endothelial, mural, and microglial cell types, likely due to their lower nuclei count (endothelial, 805 nuclei; mural, 503 nuclei; microglia, 370 nuclei; Extended Data Fig. 1-3). Nevertheless, DEGs were still identified in endothelial and mural cells when comparing 3 versus 0 h PMI samples, although no DEGs were observed in microglia (Extended Data Fig. 3-1).

Collectively, these findings suggest that while typical snRNAseq quality control metrics may be unaffected by PMI, changes in gene expression are still induced by delayed tissue banking or processing, particularly in neurons.

### Postmortem interval dampens tauopathy-specific disease signatures in neurons

Since human brain samples are central to studies of disease, we next investigated how disease-specific signatures are altered by PMI. Using pseudobulk DESeq2 differential expression analysis, we identified 49 upregulated and 146 downregulated differentially expressed genes (DEGs; *q* value <0.1) in PS19 neurons at the 0 h PMI, constituting a disease-specific neuronal signature (Fig. 4*A*). These upregulated genes were enriched in pathways related to nonsense-mediated decay, protein homo-oligomerization, neuronal system, and endocytosis (Extended Data Fig. 4-1), which are typically associated with enhanced cellular stress responses and synaptic function commonly observed in tauopathy models like PS19 (Siano et al., 2021). Conversely, the downregulated genes were associated with the RAF/MAP kinase cascade, head development, and neuron projection development (Extended Data Fig. 4-1), pathways expected to be downregulated due to impaired neuronal signaling and structural integrity in Alzheimer's disease and related tauopathies (Siano et al., 2021). Interestingly, at 3 h postmortem, this disease-specific transcriptional signal was significantly attenuated, with only 3 upregulated and 8 downregulated genes (Fig. 4*B*). Similarly, the number of DEGs between PS19 versus WT within interneurons was reduced from 135 (30 upregulated, 105 downregulated; Fig. 4*D*) to 6 (2 upregulated, 4 downregulated; Fig. 4*E*). For astrocytes, the 0 h only comparison showed 7 upregulated and 19 downregulated DEGs (Fig. 4*G*), while at 3 h, only 1 upregulated gene was detected, with no downregulated genes (Fig. 4*H*). Despite the 3 h PMI group including two more samples than the 0 h group, there was a general reduction in the detection of differentially expressed genes across all cell types, with the exception of mural cells and OPCs, where 8 and 1 more DEGs were observed at 3 h PMI *q* value <0.1, respectively. The reduction in detected DEGs at 3 h PMI underscores the challenges of identifying biological differences due to disease conditions at extended PMI, highlighting the critical importance of sample preservation time in transcriptomic studies.

10.1523/ENEURO.0505-24.2025.f4-1 Figure 4-1 **Gene ontology (GO) enrichment analysis of upregulated and downregulated genes in PS19 versus WT at 0-hour and 3-hour post-mortem intervals (PMI). A)** In the 0-hour PMI group, upregulated genes in PS19 neurons compared to WT are enriched in pathways such as nonsense-mediated decay, protein homooligomerization, steroid metabolism, neuronal systems, and endocytosis. The x-axis represents the GO term, and the y-axis, with the bar color indicating the −log10 p-value. **B)** Downregulated genes are enriched in pathways related to the RAF/MAP kinase cascade, head development, and cell cycle regulation. **C)** At 3-hour PMI, the overall signal of differentially expressed genes (DEGs) in PS19 versus WT was reduced, and no pathways were identified as upregulated due to an insufficient number of DEGs (q-value < 0.1). **D)** Downregulated genes at 3-hour PMI were enriched in supramolecular fiber organization. Download Figure 4-1 TIF file.

## Discussion

In this study, we sought to systematically evaluate cell type-specific transcriptional changes induced by postmortem interval using single-nucleus RNA sequencing from C57BL/6J wild-type (WT) and tau P301S (PS19) mouse brains. Despite delayed tissue processing at a 3 h PMI, we observed stability of basic quality control metrics, cell type proportions, and total nuclei counts across all samples, regardless of PMI or genotype. We additionally observed that neurons and interneurons exhibit a significant upregulation of genes involved in immune response and stress pathways at 3 h postmortem compared with a 0 h prepared samples. This observation aligns with previous findings that neuronal cells are particularly sensitive to postmortem conditions (Dachet et al., 2021), which may be attributed to their high metabolic demands and increased vulnerability to oxidative stress. In contrast, non-neuronal cells, such as astrocytes, primarily showed alterations in cell–cell adhesion processes, which may reflect postmortem shifts in the cellular environment. These changes are particularly relevant for studies investigating neuroinflammatory processes in neurodegenerative diseases, where the integrity of cell–cell interactions plays a crucial role (Hernandez et al., 2021; Kim et al., 2022).

Our analysis also revealed that the differential expression signal between PS19 versus WT mice diminishes at a 3 h PMI. Specifically, disease-specific transcriptomic signatures in neurons and interneurons were less pronounced in the 3 h compared with 0 h PMI samples. This finding underscores the potential for PMI to obscure disease-relevant signals, a critical consideration for studies using banked brain tissues with variable PMIs.

In conclusion, the findings emphasize the importance of considering PMI in experimental designs and data interpretation when using postmortem brain samples. Studies that do not account for PMI-related alterations risk misidentifying gene expression changes as disease-related when they may be artifacts of the postmortem process. Additionally, our results suggest that different cell types respond differently to postmortem conditions, highlighting the necessity of cell type-specific analyses in neurodegenerative studies. Future research should focus on elucidating the molecular mechanisms driving these cell type-specific responses and explore strategies to mitigate the effects of PMI. Such methods include optimizing tissue preservation techniques or incorporating PMI as a covariate in differential expression analyses.

### Limitations

This study has several limitations that should be considered when interpreting the findings. The uncertainty about whether the observed transcriptional changes across different postmortem intervals are due to biological responses or are reflective of RNA degradation presents a significant challenge. This distinction is crucial, especially in brain banking scenarios where PMI can vary widely, potentially confounding the results. To address this, future studies should include RNA integrity assessments and analyze multiple PMIs to understand better the impact of tissue processing delay on gene expression. Additionally, this study only tested a single PMI, limiting the ability to assess temporal trends in transcriptional changes. Another limitation is the underrepresentation of specific cell types, as neurons are the most prevalent cell type in single-nucleus brain data, which may skew the findings. Finally, the study was underpowered to detect potential sex differences, limiting the generalizability of the results across male and female samples.

## Synthesis

Reviewing Editor: Laura Volpicelli-Daley, The University of Alabama at Birmingham Decisions are customarily a result of the Reviewing Editor and the peer reviewers coming together and discussing their recommendations until a consensus is reached. When revisions are invited, a fact-based synthesis statement explaining their decision and outlining what is needed to prepare a revision will be listed below. The following reviewer(s) agreed to reveal their identity: Fabien Dachet.

Many research funders are requiring that postmortem human brain tissue be used to confirm results of animal models. Therefore, having the highest quality brain tissue with the lowest postmortem interval is necessary. This manuscript reviews the needs for PMI of three hours or less. This is an important manuscript for the field. A few corrections were suggested by the reviewer which should be addressed.

Line 47:Include information about the typical life expectancy of mice and clarify what human age corresponds to a 37-week-old mouse. While the context suggests it represents an older human age, this is not immediately apparent.

Line 48:Provide a brief definition of tauopathy, emphasizing its characteristics and its connection to Alzheimer's disease. e.g.: Tauopathies are neurodegenerative disorders characterized by abnormal aggregation of tau protein, with Alzheimer's disease being a well-known example.

Line 54+:Relocate any detailed experimental or procedural information currently in the Introduction to the Methods section to maintain clarity and alignment with typical journal standards.

Line 57:The discussion about the quality of DESeq2 is unnecessary and could be removed to streamline the text.

Line 63+:Results are being presented at the end of the Introduction. Verify whether the journal's guidelines permit this; otherwise, move this information to the Results section.

Line 70:Clarify what is meant by "PS19 mice were maintained on a pure B6J background after 72 backcrossings to C57Bl/6J mice"

Line 79:Instead of simply stating, whole mice were placed intact at 4{degree sign}C, specify that this refers to the corpses of euthanized mice. This will enhance clarity and improve the reading flow.

Line 153:Details about how the models were written in the code are unnecessary. Additionally, you wrote twice the same formulae :" ~ PMI + genotype + sex " and " ~ genotype + PMI + sex".

Line 295:Consider including a quick assessment of RNA quality, such as using a Bioanalyzer or a simple agarose gel electrophoresis. If sufficient RNA remains, this could be added in a revised version of the paper. Previous studies have assessed postmortem RNA quality and reported no significant degradation.

## Author Response

Computational Neuroscience Model Code Accessibility Comments for Author (Required):

Many research funders are requiring that postmortem human brain tissue be used to confirm results of animal models. Therefore, having the highest quality brain tissue with the lowest postmortem interval is necessary. This manuscript reviews the needs for PMI of three hours or less. This is an important manuscript for the field. A few corrections were suggested by the reviewer which should be addressed.

Synthesis Statement for Author (Required):

Many research funders are requiring that postmortem human brain tissue be used to confirm results of animal models. Therefore, having the highest quality brain tissue with the lowest postmortem interval is necessary. This manuscript reviews the needs for PMI of three hours or less. This is an important manuscript for the field. A few corrections were suggested by the reviewer which should be addressed.

We thank the reviewers for taking the time to review our revisions and for their thoughtful feedback.

Line 47:Include information about the typical life expectancy of mice and clarify what human age corresponds to a 37-week-old mouse. While the context suggests it represents an older human age, this is not immediately apparent.

We have added the following text to the manuscript:

Lines 49-51: The median lifespan of these mouse models is 27-29 months; thus, a 37-week-old mouse corresponds roughly to a 35-year-old human, representing mature adulthood.

Line 48:Provide a brief definition of tauopathy, emphasizing its characteristics and its connection to Alzheimer's disease. e.g.: Tauopathies are neurodegenerative disorders characterized by abnormal aggregation of tau protein, with Alzheimer's disease being a well-known example.

We have added the following text to the manuscript:

Lines 48-49: and tau P301S (PS19) mice, a model of tauopathy commonly used in Alzheimer's disease studies (Iba et al., 2013; Yoshiyama et al., 2007). Tauopathies are neurodegenerative disorders characterized by abnormal tau protein aggregations that impair normal neuronal function.

Line 54+:Relocate any detailed experimental or procedural information currently in the Introduction to the Methods section to maintain clarity and alignment with typical journal standards.

We have removed the following text from the introduction:

In addition to Seurat, we employed DESeq2 package in our analysis to better assess the robustness of post-mortem effects. DESeq2, used for pseudobulk analysis, aggregates counts across cells to mitigate the variability introduced by inter-individual differences (Love et al., 2014). In our PMI study, we identify neurons as a cell population uniquely vulnerable to post-mortem transcriptomic alterations and identify a set of 132 neuronal genes that are dysregulated in response to a 3-hour PMI in healthy, non-transgenic mice. Furthermore, we demonstrate that the neuronal disease signature in PS19 tauopathy mice is partially lost 3 hours post-mortem, suggesting samples banked or processed long after death may not fully represent the true extent of disease signatures.

The following text was moved from the introduction the methods:

Lines 147-150: Prior studies have shown varied performance across differential expression tools, highlighting a trade-off between detecting true positives and maintaining precision. DESeq2 pseudobulk was repeatedly reported as being well balanced and robust for small sample size (Jaakkola et al., 2017; Li et al., 2023; Murphy &Skene, 2022; Squair et al., 2021; Wang et al., 2019; Zimmerman et al., 2021).

Line 57:The discussion about the quality of DESeq2 is unnecessary and could be removed to streamline the text.

We removed the text in the introduction to the methods section. Since MAST is a common tool of choice for single cell differential expression analysis, we find it important to highlight why we choose to use DESeq2.

The following text was moved from the introduction the methods:

Lines 147-150: Prior studies have shown varied performance across differential expression tools, highlighting a trade-off between detecting true positives and maintaining precision. DESeq2 pseudobulk was repeatedly reported as being well balanced and robust for small sample size (Jaakkola et al., 2017; Li et al., 2023; Murphy &Skene, 2022; Squair et al., 2021; Wang et al., 2019; Zimmerman et al., 2021).

Line 63+:Results are being presented at the end of the Introduction. Verify whether the journal's guidelines permit this; otherwise, move this information to the Results section.

We have removed the following text from the introduction:

In addition to Seurat, we employed DESeq2 package in our analysis to better assess the robustness of post-mortem effects. DESeq2, used for pseudobulk analysis, aggregates counts across cells to mitigate the variability introduced by inter-individual differences (Love et al., 2014). In our PMI study, we identify neurons as a cell population uniquely vulnerable to post-mortem transcriptomic alterations and identify a set of 132 neuronal genes that are dysregulated in response to a 3-hour PMI in healthy, non-transgenic mice. Furthermore, we demonstrate that the neuronal disease signature in PS19 tauopathy mice is partially lost 3 hours post-mortem, suggesting samples banked or processed long after death may not fully represent the true extent of disease signatures.

Line 70:Clarify what is meant by "PS19 mice were maintained on a pure B6J background after 72 backcrossings to C57Bl/6J mice" We add the following text to the manuscript:

Lines 63-65: We used N=9 wild-type (WT) C57BL/6J and N=9 tau P301S (PS19) mice (Iba et al., 2013; Yoshiyama et al., 2007) at 37 weeks of age in this study. PS19 mice were bred with pure C57Bl /6J background after for >10 generations to ensure that the PS19 genetic background is nearly identical to the C57Bl/6J strain, apart from the transgene (P301S tau).

Line 79:Instead of simply stating, whole mice were placed intact at 4{degree sign}C, specify that this refers to the corpses of euthanized mice. This will enhance clarity and improve the reading flow.

The text has been updated to make this point clearer:

Lines 71-73: For the 3-hour PMI group, the whole corpses of the euthanized mice were placed intact at 4{degree sign}C for three hours before subsequently removing the brain for further processing (Fig. 1A).

Line 153:Details about how the models were written in the code are unnecessary. Additionally, you wrote twice the same formulae :" ~ PMI + genotype + sex " and " ~ genotype + PMI + sex".

Details about how the model was written in the code have been removed from the text.

Line 295:Consider including a quick assessment of RNA quality, such as using a Bioanalyzer or a simple agarose gel electrophoresis. If sufficient RNA remains, this could be added in a revised version of the paper. Previous studies have assessed postmortem RNA quality and reported no significant degradation.

We thank the reviewer for the suggestion and have added RIN values to the manuscript. The RIN value for each sample is now reported in Supplementary Table 1. There is no difference in mean RIN values between PMI groups. The following text has been added to the manuscript:

Lines 83-86 of the methods section: Total RNA was extracted from frozen mouse brain tissue using the Qiagen RNeasy Plus Mini Kit, following the manufacturer's protocol. RNA integrity was assessed on an Agilent Tapestation using the RNA ScreenTape kit to obtain RINe values (Supplemental Table 1-1).

Lines 174-175 of the results section: We first investigated RINe values to determine if a delayed PMI resulted in lower RIN values. There was no difference in mean RINe values between fresh 0-hour and 3-hour PMI (t(16) = 0.893, p = 0.385).
