# Supplementary figures and images for: Postmortem Interval Leads to Loss of Disease-Specific Signatures in Brain Tissue
Source: eNeuro. 2025 Mar 7;12(3):ENEURO.0505-24.2025. doi: 10.1523/ENEURO.0505-24.2025 (PMC11913402; doi:10.1523/ENEURO.0505-24.2025)

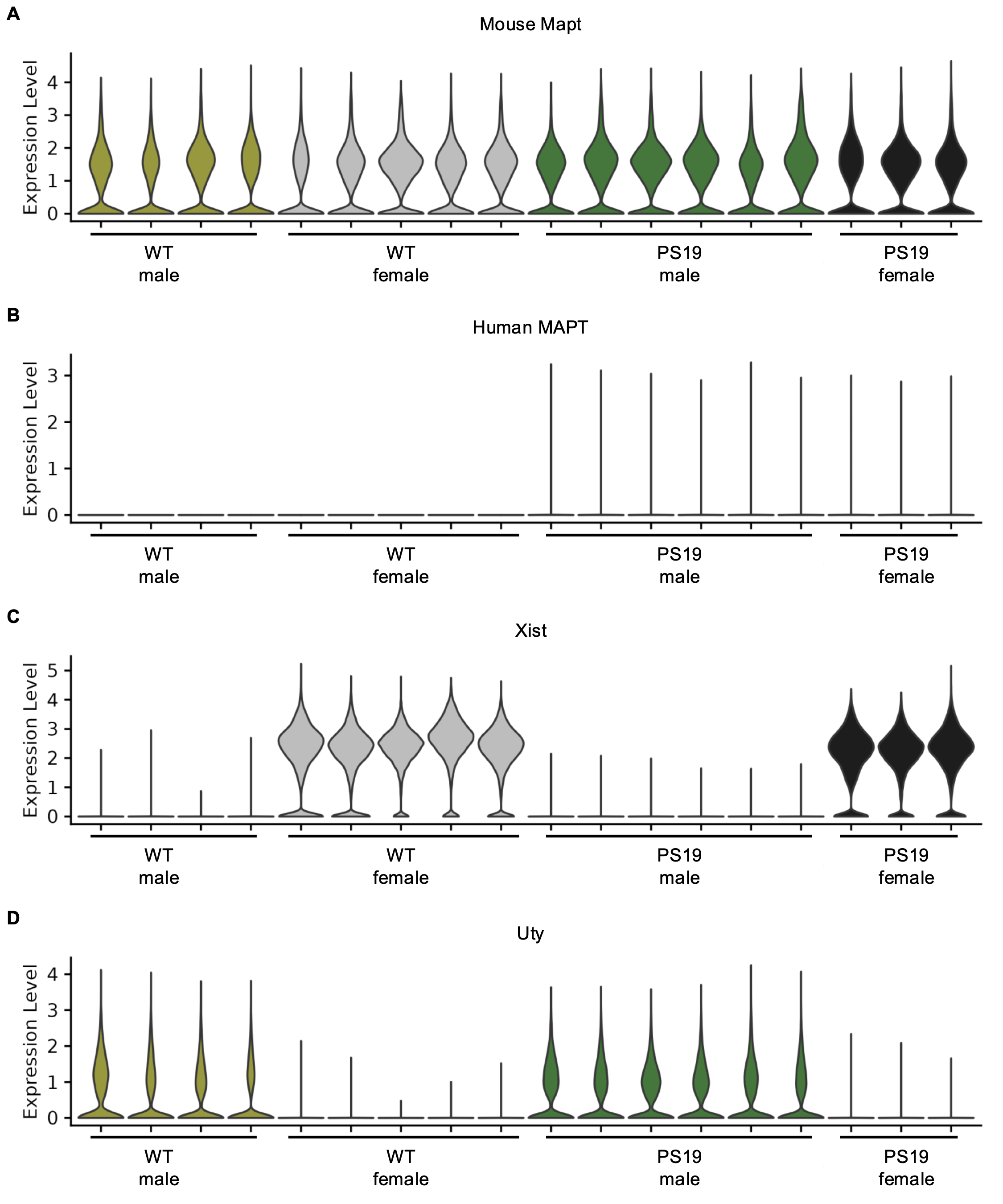

Supplement: Figure 1-1 — Sample genotype and sex verification. Log normalized gene expression of A) mouse Mapt, B) human MAPT, mouse C) Xist, and D) Uty. Download Figure 1-1 TIF file. [file eneuro-12-ENEURO.0505-24.2025-s002.tif]

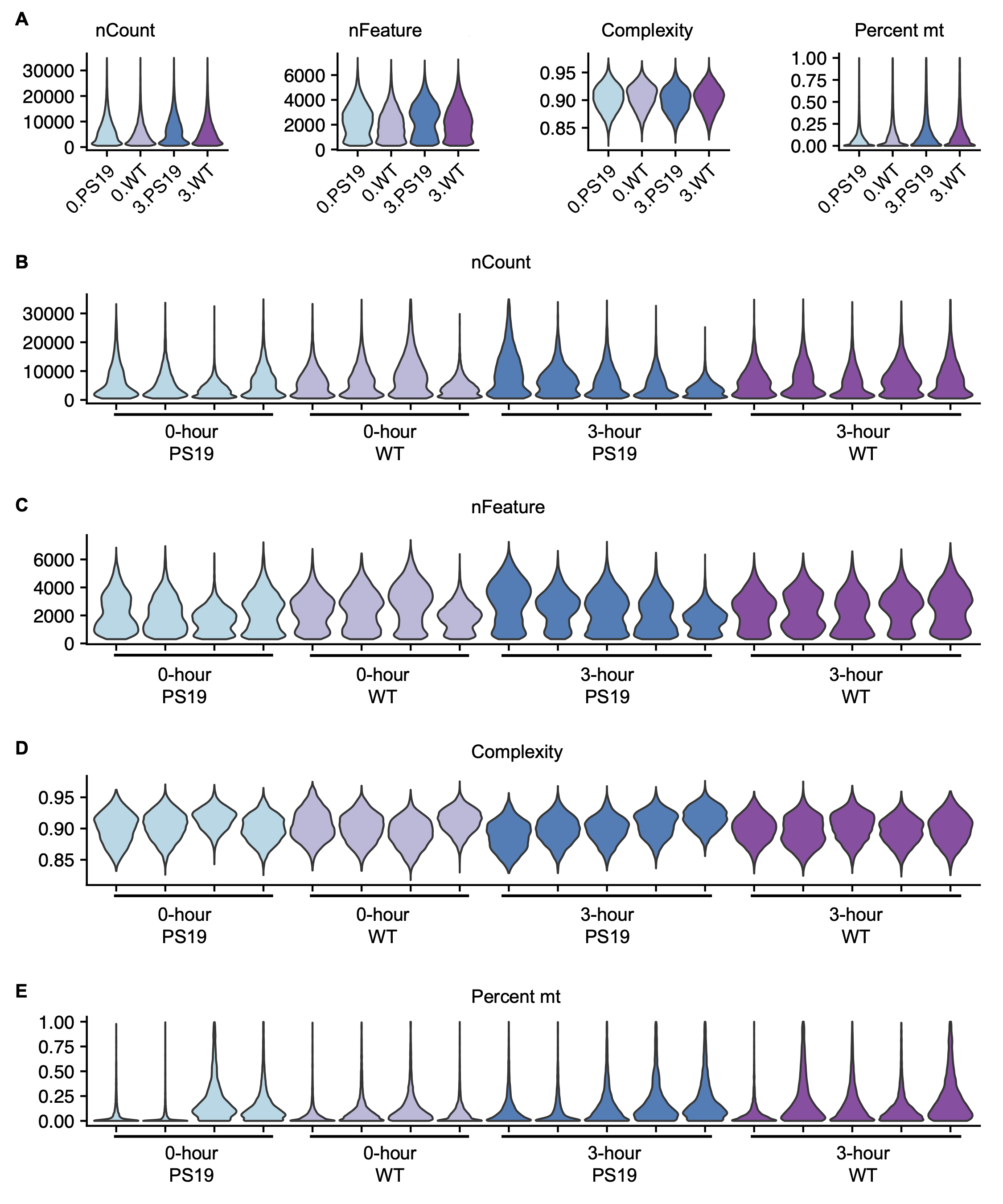

Supplement: Figure 1-2 — Single nucleus quality metrics do not differ among mice. A) Violin plots illustrating primary quality control (QC) metrics, including the number of counts per nucleus (nCount), detected features per nucleus (nFeature), cell complexity (the log10 ratio of nFeature to nCount), and the percentage of mitochondrial gene expression (percent mt) for PS19 0-hour (N = 4), WT 0-hour (N = 4), PS19 3-hour (N = 5), and WT 3-hour (N = 5) mice. Quality control metrics for each mouse for B) nCount, C) nFeature, D) cell complexity, and E) percent mt. Download Figure 1-2 TIF file. [file eneuro-12-ENEURO.0505-24.2025-s003.tif]

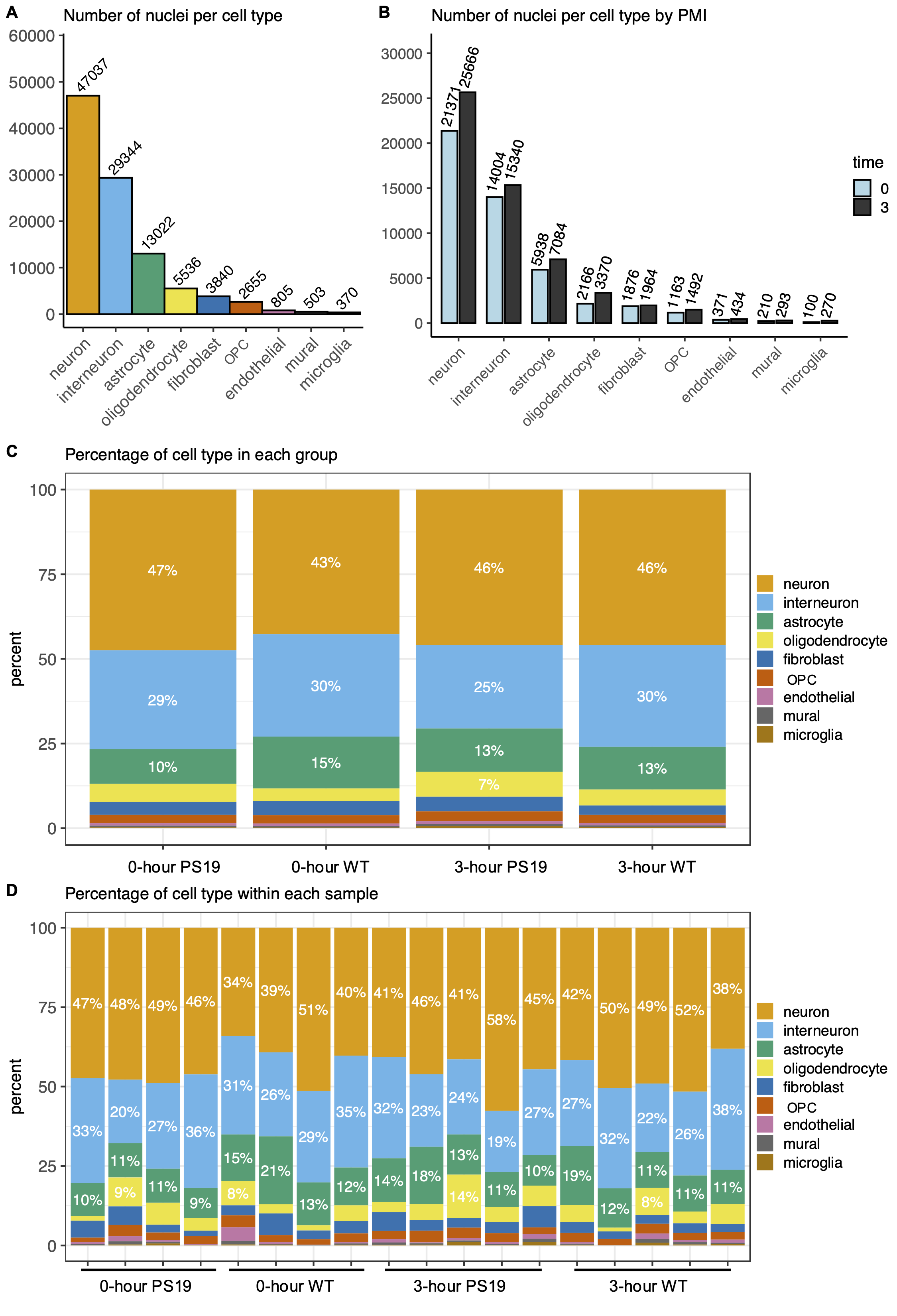

Supplement: Figure 1-3 — Relative abundance of cell types among samples. A) the total nuclei count for each cell type is shown for both the 0-hour and 3-hour post-mortem interval (PMI) sample groups. B) the percentage of each cell type is presented across different genotypes (PS19 or WT) and PMI durations (0-hour or 3-hour). C) The proportion of each cell type within individual samples is illustrated, providing a detailed view of the distribution across all samples. Download Figure 1-3 TIF file. [file eneuro-12-ENEURO.0505-24.2025-s004.tif]

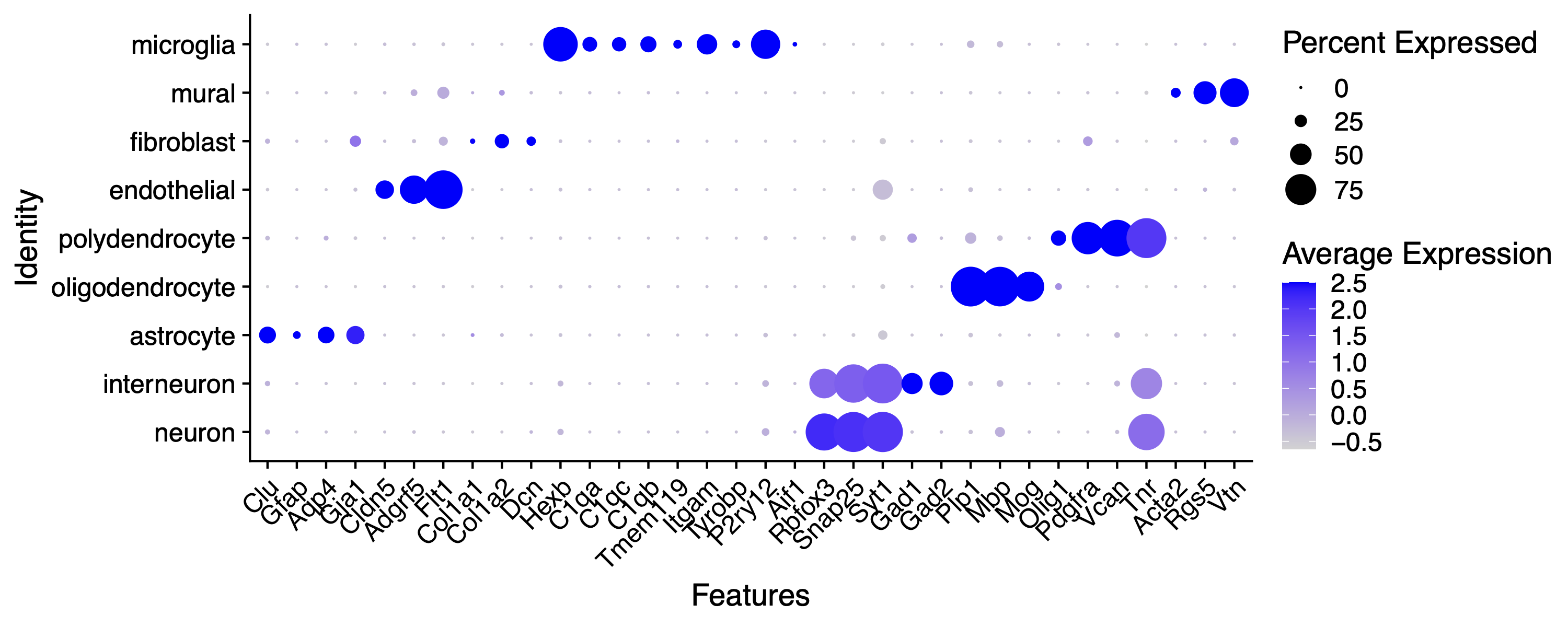

Supplement: Figure 1-4 — Bubble plot showing brain cell type markers. The Y-axis represents different cell types, while the X-axis displays the corresponding biomarker genes. The size of each bubble indicates the percentage of nuclei within a given cell type that expresses that gene and the color represents the average log2 expression level. Download Figure 1-4 TIF file. [file eneuro-12-ENEURO.0505-24.2025-s005.tif]

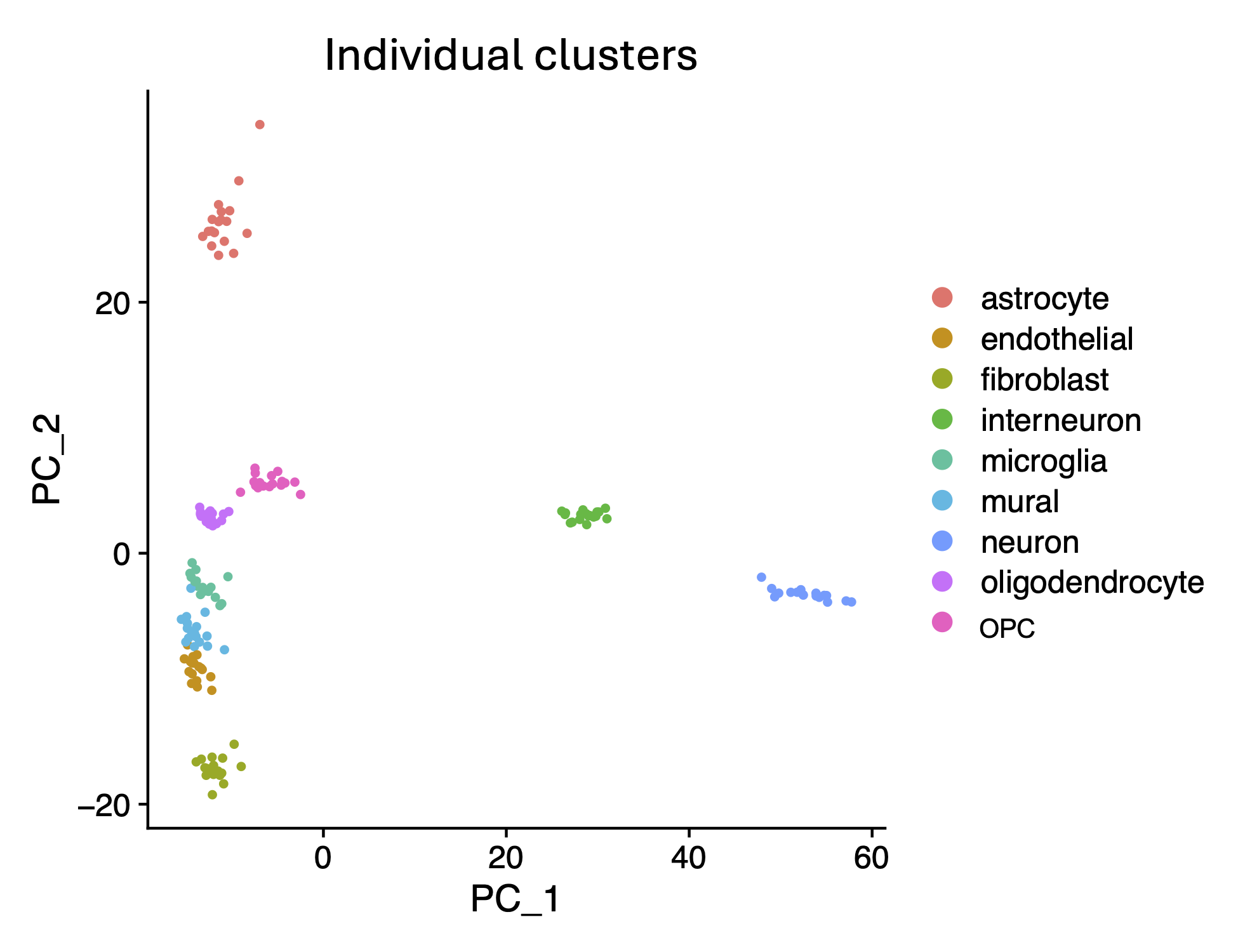

Supplement: Figure 1-5 — Principal component analysis (PCA) of aggregated expression values at the sample and cell type level. Clear delineation of the major cell types. Download Figure 1-5 TIF file. [file eneuro-12-ENEURO.0505-24.2025-s006.tif]

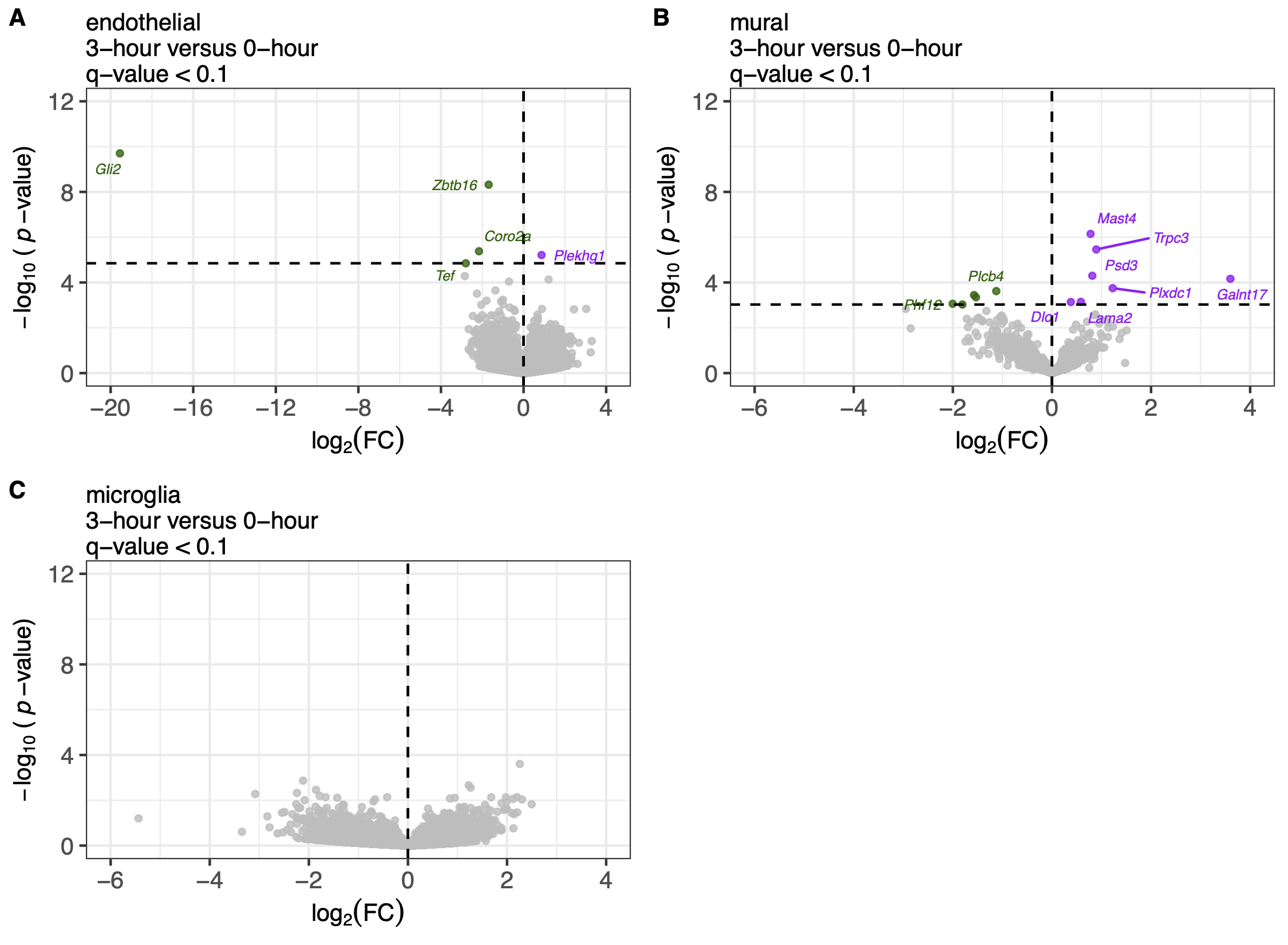

Supplement: Figure 3-1 — The transcriptomic effect of PMI 3-hour versus 0-hour PMI for endothelial, mural, and microglia cell types. A) Volcano plot of differentially expressed genes (DEGs) in endothelial cell type, comparing 3-hour PMI (N = 10) versus fresh 0-hour (N = 8) samples (total N = 18). Downregulated genes (log2 fold change < 0, q < 0.1) are shown in green, upregulated genes (log2 fold change > 0, q < 0.1) in purple, and non-significant genes (q ≥ 0.1) in gray. Repeated for B) mural and C) microglia. Download Figure 3-1 TIF file. [file eneuro-12-ENEURO.0505-24.2025-s007.tif]

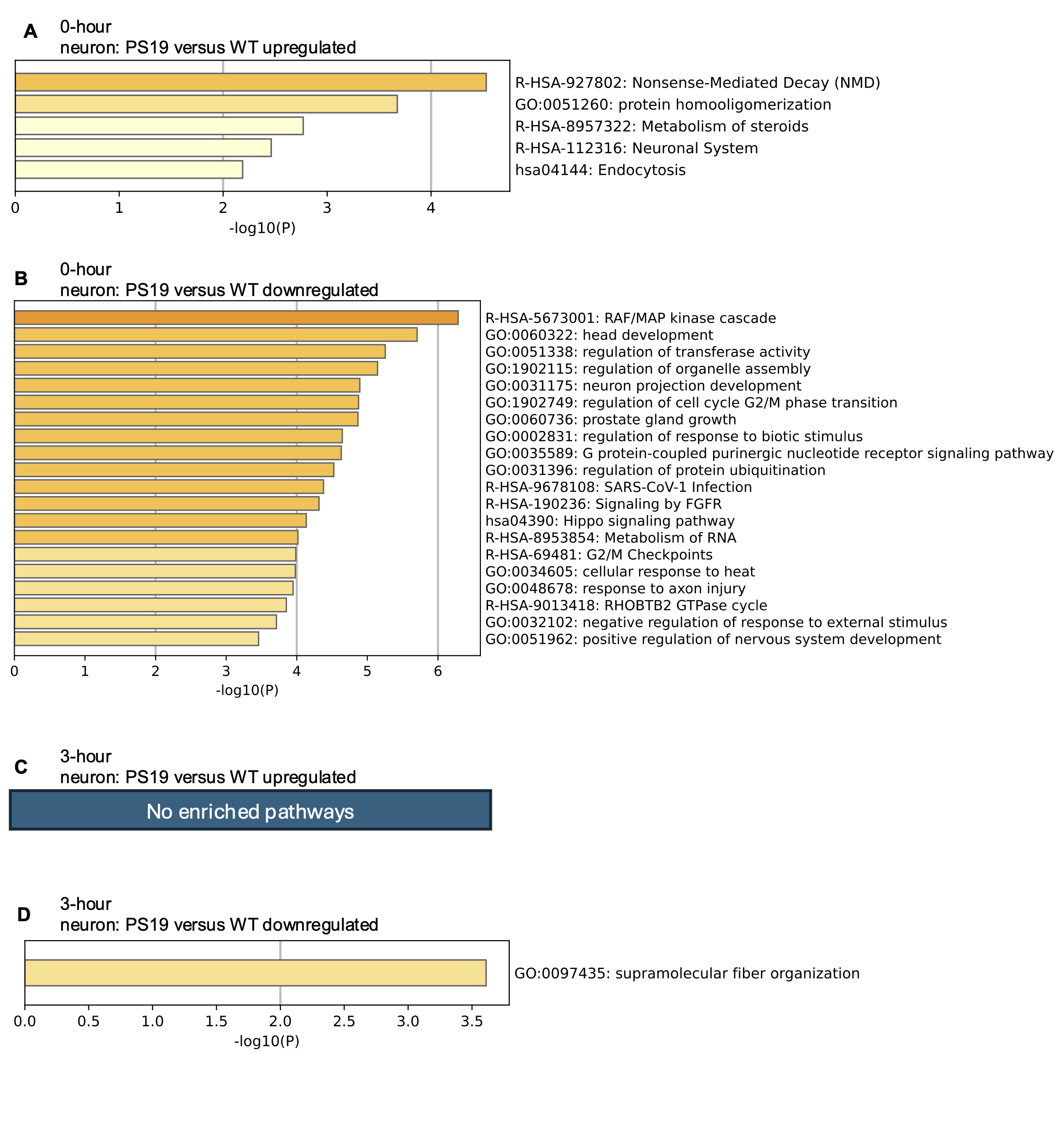

Supplement: Figure 4-1 — Gene ontology (GO) enrichment analysis of upregulated and downregulated genes in PS19 versus WT at 0-hour and 3-hour post-mortem intervals (PMI). A) In the 0-hour PMI group, upregulated genes in PS19 neurons compared to WT are enriched in pathways such as nonsense-mediated decay, protein homooligomerization, steroid metabolism, neuronal systems, and endocytosis. The x-axis represents the GO term, and the y-axis, with the bar color indicating the −log10 p-value. B) Downregulated genes are enriched in pathways related to the RAF/MAP kinase cascade, head development, and cell cycle regulation. C) At 3-hour PMI, the overall signal of differentially expressed genes (DEGs) in PS19 versus WT was reduced, and no pathways were identified as upregulated due to an insufficient number of DEGs (q-value < 0.1). D) Downregulated genes at 3-hour PMI were enriched in supramolecular fiber organization. Download Figure 4-1 TIF file. [file eneuro-12-ENEURO.0505-24.2025-s008.tif]
