# Supplementary material for: Postmortem Interval Leads to Loss of Disease-Specific Signatures in Brain Tissue
Source: eNeuro. 2025 Mar 7;12(3):ENEURO.0505-24.2025. doi: 10.1523/ENEURO.0505-24.2025 (PMC11913402; doi:10.1523/ENEURO.0505-24.2025)
Supplement: Table 1-1 — Metadata. Sample ID, genotype PS19 tau or wildtype (WT), sex, post-mortem interval (PMI) in hours, and RNA integrity number (RINe). Download Table 1-1. Metadata., DOCX file. [file eneuro-12-ENEURO.0505-24.2025-s001.docx]

**Table 1-1. Metadata.** Sample ID, genotype PS19 tau or wildtype (WT), sex, post-mortem interval (PMI) in hours, and RNA integrity number (RIN^e^).

|  | **Sample ID** | **Genotype** | **Sex** | **PMI in hours** | **RIN^e^** |
| --- | --- | --- | --- | --- | --- |
| 1 | J_3 | WT | Male | 0 | 9.1 |
| 2 | J_4 | WT | Male | 0 | 9.4 |
| 3 | B_7 | WT | Female | 0 | 9.2 |
| 4 | P_65 | WT | Female | 0 | 9.3 |
| 5 | P_52 | PS19 | Male | 0 | 9.1 |
| 6 | P_62 | PS19 | Male | 0 | 9.4 |
| 7 | P_67 | PS19 | Male | 0 | 9.2 |
| 8 | P_69 | PS19 | Female | 0 | 9.5 |
| 9 | J_1 | WT | Male | 3 | 9.6 |
| 10 | J_2 | WT | Male | 3 | 9.1 |
| 11 | B_10 | WT | Female | 3 | 9.0 |
| 12 | B_8 | WT | Female | 3 | 9.2 |
| 13 | B_9 | WT | Female | 3 | 9.2 |
| 14 | P_53 | PS19 | Male | 3 | 9.3 |
| 15 | P_54 | PS19 | Male | 3 | 9.2 |
| 16 | P_64 | PS19 | Male | 3 | 9.3 |
| 17 | P_68 | PS19 | Female | 3 | 9.3 |
| 18 | P_71 | PS19 | Female | 3 | 8.7 |
